# Understanding the Motivation of Western Java Smallholder Broiler Farmers to Uptake Measures Against Highly Pathogenic Avian Influenza (HPAI)

**DOI:** 10.3389/fvets.2020.00362

**Published:** 2020-07-21

**Authors:** Muchammad Gumilang Pramuwidyatama, Henk Hogeveen, Helmut W. Saatkamp

**Affiliations:** ^1^Business Economics Group, Wageningen University & Research, Wageningen, Netherlands; ^2^Department of Farm Animal Health, Faculty of Veterinary Medicine, Utrecht University, Utrecht, Netherlands

**Keywords:** poultry farmer, theory of planned behavior, vaccination, biosecurity, highly pathogenic avian influenza, endemic, small-scale, HPAI

## Abstract

Understanding broiler farmers' intention toward highly pathogenic avian influenza (HPAI) control is important to design successful HPAI control programs. We used Theory of Planned Behavior (TPB) to identify factors (i.e., attitude, subjective norm, and perceived behavioral control) associated with the intentions of Western-Java small-scale broiler farmers toward implementing cleaning and disinfection (C&D), vaccination, reporting, and stamping-out without or with 50% compensation. For this, 203 Western-Java farmers were interviewed. The majority of the farmers had a positive intention to implement C&D (89%), reporting (88%), and vaccination (80%). A lower number had a positive intention to join stamping-out both with 50% compensation (67%) and without any compensation (53%). Farmers had a more positive attitude and subjective norm, but lower perceived behavioral control toward one or more of the intentions to implement measures. Attitude was positively associated with intentions to implement C&D and vaccination. Subjective norm of veterinarians of integrated companies was positively associated with intentions to implement vaccination. Perceived behavioral control (i.e., money and time) was positively associated with intentions to implement C&D, vaccination, and stamping-out without any compensation. Results suggest that farmers are in favor of implementing preventive measures (i.e., C&D and vaccination) on HPAI control over facing the consequences of control measures (i.e., stamping-out), and HPAI control programs should primarily focus on incentivizing farmers complemented by programs aiming to improve farmers' attitude. Thus, policy should be emphasized to preventive measures rather than control measures. Financial incentive-based instruments (e.g., price and performance bonus) can be used to increase the intention of farmers to implement C&D and vaccination. Trained vaccinators might help to save the time needed to vaccinate the entire flock can increase the intention of farmers to vaccinate their chickens. Also, informational instruments (e.g., education and communication) can be used to change and to improve the attitude of farmers to implement both measures.

## Introduction

Highly pathogenic avian influenza (HPAI) is a zoonotic disease that severely infects both poultry and humans and has a high mortality rate [(1), p. 247]. Highly pathogenic avian influenza has had severe consequences in Indonesia. During a major HPAI outbreak in 2003–2004, many small-scale poultry farmers stopped their farming activities and, as a result, lost their primary source of income [(2), p. 7–8]. Furthermore, there were 200 reported human cases of HPAI leading to 168 casualties (3). Highly pathogenic avian influenza has remained endemic in most Indonesian regions. The number of reported outbreaks of avian influenza (AI) in 2018 reduced to 476, which is five times lower than that in 2007 (4). However, the actual number of outbreaks could be higher because many cases go unreported [(5), p. 8]. Based on this enormous impact, HPAI has been declared a national priority zoonotic disease by the national government since 2005 (6).

Highly pathogenic avian influenza is of particular importance for Western Java, because it has both the largest human and broiler chicken population in the country, accounting for 29% and 6% of the national populations, respectively (7). Highly pathogenic avian influenza has also remained endemic in Western Java to varying degrees across regencies and districts. Local governmental agencies in Western Java control HPAI based on the national HPAI control strategy comprised nine measures: [1] improvement of biosecurity, [2] selective depopulation, [3] vaccination, [4] traffic control, [5] surveillance and monitoring, [6] increasing public awareness, [7] poultry restocking, [8] stamping-out, and [9] monitoring and evaluation (8). Three of these measures are targeted at farms: improvement of biosecurity, routine AI vaccination in an endemic district, and reporting (8). Improvement of biosecurity of broiler farms and AI vaccination have been top-priority programs of the government (9). Stamping-out as a control measure is currently used only in newly infected districts, whereas selective depopulation of infected chickens is implemented in endemic districts. However, the implementation of these control measures has been incomplete and ineffective because of poor infrastructure, the complex structure of the poultry sector, poor incentives for farmers, and budget limitations [(9), p. 1–2]. While biosecurity and vaccination could be implemented by farmers themselves and can even be economically beneficial for farmers, there has especially been a low uptake of HPAI control measures among small-scale commercial and backyard broiler farmers.

It is clear that farmers' behavior is important in the control of HPAI. Currently, there is a lack of understanding of what factors influence the decision of farmers to take up measures against HPAI. Assuming that increased uptake of HPAI control measures among small-scale broiler farmers will aid HPAI mitigation, understanding the factors that influence their motivation to do so is important. Understanding the drivers of broiler farmers is necessary to design HPAI mitigation schemes that are efficient and effective because they have a high likelihood of adoption by farmers. To date, studies on this topic were focused exclusively on sociodemographic characteristics of farmers (10, 11) and farm characteristics (12). A recent study by Indrawan et al. (13) evaluated farmers' characteristics and business types in relation to the implementation of biosecurity measures on broiler farms in Western Java. However, the decision to implement measures against HPAI cannot be explained by sociodemographic and farm characteristics alone. Ajzen (14), through the Theory of Planned Behavior (TPB), states that sociopsychological factors, such as attitude (AT), subjective norms (SN), and perceived behavioral control (PBC), also determine the uptake of a particular action. The TPB states that the intention to perform a behavior is the best predictor of actual behavior (10, p. 179). The TPB has been applied in several studies to gain insight into the psychological factors that influence intentions to take up measures related to animal disease control. Examples include the uptake of rabies vaccination by Indonesian dog owners (15), the uptake of biosecurity measures by dairy cattle farmers in Great Britain (16), and mastitis control by Ethiopian dairy farmers (17). However, no study has used the TPB to evaluate risk mitigation for HPAI. Applying a behavior-explaining framework might shed light on psychological factors of the willingness of farmers to take up different measures against HPAI and consequently might help their implementation.

This article aims to identify (1) if and how psychological factors (i.e., AT, SN, and PBC) of farmers in Western Java are associated with their intention to implement different measures against HPAI and (2) sociodemographic characteristics that affect the farmers' intentions through relations with their AT, SN, and PBC.

## Materials and Methods

### Theoretical Framework

**Figure 1:**
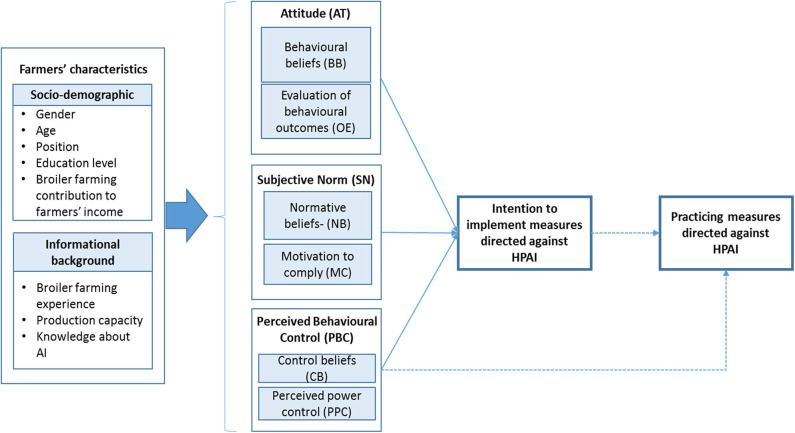
Theory of planned behavior framework for the intention to implement measures against HPAI adapted from Ajzen (14). Dotted lines indicate relationships which are not studied in this article.

According to the TPB (Figure 1), behavioral intention is the best prediction of future behavior (i.e., to perform or not to perform a certain behavior) [(14), p. 179–180]. The theory proposes that a behavioral intention is determined by three psychological factors, namely, AT, SN, and PBC, as shown in Figure 1 [(14), p. 179–180].

When applying the TPB to the context of changing farmers' behavior regarding HPAI control, AT can be defined as a farmer's beliefs about the outcomes of performing measures against HPAI [i.e., behavioral beliefs (BBs)] weighted by their evaluation of these outcomes [i.e., outcome evaluation (OE)]. For example, farmers who strongly believe and highly value the outcomes of vaccination are expected to have a more positive AT toward vaccination.

Similarly, SN can be defined as a farmer's beliefs on social pressure or other people's opinions about the implementation of HPAI prevention and control measures on their farms (i.e., normative beliefs) weighted by their motivation to comply (MC) with these pressures or opinions (i.e., MC). Influential opinions could originate from technical support (TS) and veterinarians of the integrated company, government veterinarians, TS of animal medicine companies, buyers or customers, broiler farmer peers, family members, neighbors or friends, people who live nearby, and role-model farmers.

Finally, PBC can be defined as a farmer's beliefs about factors such as money, time, and skills required to implement HPAI measures [i.e., control beliefs (CBs)], weighted by their confidence in the power of each control factor to facilitate or inhibit the decision to implement the measures [i.e., perceived power of control (PPC)].

### Measures Against HPAI at the Farm Level

This article studies the intention of small-scale commercial broiler farmers to implement any of the following preventive, monitoring, and control measures against HPAI: [1] improved biosecurity [i.e., routine cleaning and disinfection (C&D) of the farm area or barn], [2] vaccination, [3] reporting, and [4] stamping-out.

Biosecurity is defined as a set of isolation and sanitation measures with the aim of preventing the introduction as well as the spreading of diseases on the farm. Biosecurity measures will reduce the general risk of avian disease introduction, including HPAI. Ensuring or maintaining the sanitation of the farm, barn, and equipment is recognized as one of the appropriate and practical biosecurity measures on poultry farms (18, 19). In this study, sanitation measures, defined as routine C&D of the farm area or barn for every 2 days, are used as a proxy for biosecurity measures. The term *biosecurity* was not used in interviews with farmers because the term is not well-recognized and is interpreted differently among farmers. Avian influenza vaccination is defined as the implementation of AI vaccination of 7-day-old chickens via subcutaneous injection. This definition was tailored to the Western Java context in which having chickens slaughtered in multiple batches in every rearing cycle is a common practice. Farmers usually start selling their chickens when they are 21–25 days old, and AI vaccines require 14 days to provide sufficient protection from HPAI [(20), 146, p. 18,145–18,146].

Reporting is defined as declaring the observation of HPAI symptoms in one or more chickens to the authority or TS. Surveillance is based on participatory disease surveillance and response due to the lack of surveillance capacity of the veterinary and laboratory services [(21), p. 750]. In theory, reporting is suggested to be an effective early detection tool [(22), p. 435].

Stamping-out is an effective way to eradicate the virus at the source and to prevent further spreading. We included stamping-out in this study because it has been suggested to be effective and efficient in regions with low HPAI endemicity, although the program has been terminated since 2007 [(21), p. 752]. In this study, two scenarios of stamping-out were used, namely, with and without 50% compensation for culled healthy chickens. As such, the approach is adapted to the conditions of limited budgets for HPAI mitigation in Indonesia.

These four measures can be further categorized into two groups based on their scope. Biosecurity and vaccination can be seen as measures that are more in line with the prime interest of farmers for their farm, whereas reporting and stamping-out are in the interest of the sector at large (i.e., as not to be a liability in the farmer community), the government, and the public.

### Questionnaire Design

The questionnaire was developed based on the TPB framework and the list of HPAI measures explained above. The questionnaire contained two parts. The first part collected information about farmers' sociodemographic characteristics (e.g., age, education) and farm characteristics (e.g., chicken population). The second part collected information about intentions, AT, SN, and PBC regarding the four measures defined above.

The first part of the questionnaire used multiple-choice, open-ended and closed questions to collect information related to respondents' and farm characteristics, such as age, gender, education, poultry farming experience, chicken population per cycle, awareness of HPAI and its signs, and dependency level on broiler farming. In the second part of the questionnaire, Ajzen's TACT principle, which stands for target, action, context, and time, was used to define intentions. For example, “If HPAI (target) were to occur in the environment where my farm is located (context) within 1 year (time), I would vaccinate all my chickens once in every production cycle on the seventh day (action).” The target and context were used as follows: “If HPAI were to occur in the environment where my farm is located within 1 year” in the sections of BBs, OE, MC, and CBs to emphasize the hypothetical situation to respondents. No additional phrasing was used in the sections of normative beliefs and PPC because these sections are about the opinions of referents and farmers' resources in general.

A five-point Likert scale was used in the second part of the questionnaire. Respondents were asked to state the extent of their agreement/disagreement (1 = strongly disagree, 2 = disagree, 3 = neutral, 4 = agree, 5 = strongly agree) to all statements in the sections of intentions, BBs, MC, CBs, and PPC. Respondents were also asked to state the extent of the importance (1 = very unimportant, 2 = unimportant, 3 = neutral, 4 = important, 5 = very important) of all statements in the sections of OE and normative beliefs. The option “do not know” was added to the five-point Likert scale in the normative beliefs section.

Attitude was evaluated by asking respondents about their BBs toward the outcome of implementing each HPAI measure and their subjective evaluation of the importance of different outcomes (OEs). To estimate BBs, respondents were asked about their agreement related to possible outcomes of each measure (e.g., “cleaning and disinfecting my chicken barn every 2 days will reduce the risk of HPAI infection on my farm”). Respondents were also asked to give their subjective evaluation of the importance of outcomes (e.g., “reducing the risk of AI infection on my farm is…”). The following outcomes were included in this study: reduction of HPAI introduction on the farm, prevention of HPAI infection to other chickens in the flock, reducing mortality rate in the flock, prevention of the spread of HPAI to other poultry farms, prevention of HPAI transmission to humans (i.e., high-risk groups), and increase in the likelihood of the farm to be included in stamping-out.

Subjective norms were evaluated by asking respondents about the normative beliefs (NBs) of referents on the importance of implementing HPAI prevention and control measures on their farms (e.g., “according to your knowledge, what is the opinion of your technical support about prevention and control of HPAI?”). Next, respondents were asked about their own MC to the opinions of relevant referents (e.g., “do opinions of your technical support influence you to implement prevention and control of HPAI on your farm?”). If respondents indicated they did not know the opinion of a referent in section NBs, they would not be asked about their MC to the respective referent.

Perceived behavioral control was evaluated based on the resources available to farmers (time, money, and skills) to implement HPAI measures. To measure CBs, respondents were asked to indicate whether implementing the measures is time-consuming, expensive, or difficult (e.g., “implementing HPAI vaccination once in every cycle is expensive”). Then, respondents were asked about their PPC through statements that imply whether respondents perceive they have the necessary skills, spare time, and financial resources to implement measures (e.g., “I can afford to pay the costs for implementing HPAI vaccination once in every cycle”).

The questionnaire was first written in English, then translated to Bahasa Indonesia, and translated back to English for verification and publication. The questionnaire was tested in a pilot study. Ten small-scale broiler farmers were interviewed to check their understanding of the statements, as well as their ability to answer the questionnaire. Statements and terminologies that were difficult to understand by the test-farmers were modified.

### Data Collection

#### Survey Location

The poultry sector in Western Java consists of a mix of industrialized (sectors 1 and 2), small-scale commercial (sector 3), and backyard (sector 4) farms; the latter two make up the majority of farms and are widely spread across the region [(23), p. 9]. Small-scale commercial or sector 3 broiler farms keep the chicken inside the barn all the time with low biosecurity [(23), p. 9]. Sector 3 broiler farms are usually located closed or even neighboring to other sector 3 and/or sector 4 poultry farms, as well as to the neighborhood [(5, 23), p. 13].

The survey targeted small-scale commercial broiler farmers or staff in charge of farm management. We selected four regencies as the survey locations (Bogor, Subang, Ciamis, and Tasikmalaya regencies) based on several criteria: broiler chicken population size in Western Java (24); endemic HPAI; different dominant farming schemes (contract, *makloon*, i.e., farmers are paid based on the number of chickens slaughtered, or independent); and operational and logistical factors (i.e., easy access to the regencies, districts, and farms).

Bogor is located south of Jakarta and produces ~19 million broiler chickens per year at 2,200 broiler farms. Subang is located east of Jakarta with a production of ~8 million broiler chickens per year at 700 broiler farms. Ciamis is located in the southeast of the Western Java with a production of ~14.5 million broiler chickens per year at 4,000 broiler farms. Tasikmalaya is located next to Ciamis with a yearly production of 5 million broiler chickens at 1,900 broiler farmers. Subang and Bogor regencies are the main broiler-producing regions where the majority of farms operate under a price contract farming scheme. Ciamis and Tasikmalaya regencies are important producers of broilers where the majority of farms operate under a *makloon* scheme. Currently, there is no region where independent broiler farms are the dominant scheme. They are less and less common, and many such farms have changed to either contract or *makloon* scheme over the years. Thus, we assumed that the population of independent broiler farms is the smallest in all the regencies.

#### Sampling

Stratified proportional (random) sampling was used to include sufficient respondents from each farming scheme (i.e., contract, *makloon*, and independent) in the data collection. We aimed to have a total of 200 respondents (Table 1), well-beyond the acceptable sample size of 80 with a 50% response rate [(25), p. 29]. The sample size was increased to 220 respondents to account for incomplete interviews. Because integrated companies can change their contract scheme, there are no published data about the number of farms under contract and *makloon* schemes. Thus, stratification by dominant scheme in each of the regencies was based on personal communications with Indonesian poultry experts. That study identified a mix of contract, *makloon*, and independent production systems in every regency, with some of the regencies having either contract or *makloon* as the dominant scheme. For each regency, two to three subdistricts with the highest broiler population were selected as survey locations to make sure the sampling target was achieved within the time and logistic constraints.

**Table 1:** The stratification of samples for each farming scheme in all the regencies.

**Regency**	**Contract**	**Makloon**	**Independent**	**Total**
Subang	30 (29)	10 (22)	10 (0)	50 (51)
Bogor	30 (9)	10 (36)	10 (2)	50 (47)
Tasikmalaya	10 (20)	30 (30)	10 (2)	50 (52)
Ciamis	10 (10)	30 (53)	10 (0)	50 (53)
Total	80 (58)	80 (141)	40 (4)	200 (203)

*Numbers in the brackets show the number of respondents who completed the interview during the field work*.

The survey was conducted in March 2018 over 8 days; 2 days for each regency. Two survey teams, each consisting of four enumerators, visited each regency at the same time. Each team was deployed in a different subdistrict and was assisted by government officials with knowledge of the area and the local language (i.e., Sundanese). However, the government officials did not join the interview. Upon arrival at the survey location, the enumerators spread out to visit different farms to conduct a farmer or staff member interview. In addition, snowball sampling led to additional respondents after concluding the interview or by asking people who live nearby chicken farms.

The study is exempted from ethics approval from the Social Sciences Ethics Committee of Wageningen University and Research (WUR). However, the survey complies with the rules of data collection and management in WUR and the codes of ethics for research involving human participants in Indonesia. These codes require that participants have to be well-informed about the aims of the research, as well as about the anonymity in collecting and analyzing data [also stated in (26)].

At the start of the interview, all respondents were asked for their consent. Statements were read out loud by the enumerators, and respondents were given a response sheet on which they could pinpoint their response with their finger. The enumerators recorded each response on the questionnaire sheet. All data were analyzed and reported anonymously. In total, we visited 223 small-scale broiler farms. Of these, 20 farmer interviews were not included in the study because farmers were not finished, leaving 203 farmer interviews to be included in the study.

### Statistical Analysis

The data were checked for errors and missing values. A descriptive analysis was carried out on farmers' and farm characteristics, the intention to implement the four HPAI measures, and product composites of TPB factors. Product composites were created to measure the three TPB factors (i.e., AT, SN, and PBC) with as little variables per TPB factor as possible. According to the TPB framework (Figure 1), the product composite of AT is the product of BB and the corresponding OE. The product composite of SN is the product of normative belief and the corresponding MC. The product composite of PBC is the product of CB and the corresponding PPC. Theoretically, the scores for all product composites range from 1 to 25.

For each of the measures, the internal consistency among the product composites of AT, SN, and PBC was evaluated through Cronbach α. If Cronbach α > 0.7, internal consistency among the product composite for that factor was regarded as significant (22, p. 574). For those factors, the product composites were averaged to derive a single direct measure (i.e., mean score) as shown in equations 1 to 3 [(27), p. 405]. When the product composites were inconsistent (Cronbach α < 0.7), a subset of product composites within the factor was used to find a combination of product composites that was internally consist. The other product composites were used as separate TPB factors.

(1) $$\begin{array}{l} {AT}_{i}=\sum_{j=1}^{n}\left({BB}_{j}\;x\;{OE}_{j}\right)/n \end{array}$$

(2) $$\begin{array}{l} {SN}_{i}=\sum_{j=1}^{n}\left({NB}_{j}\;x\;{MC}_{j}\right)/n \end{array}$$

(3) $$\begin{array}{l} {PBC}_{i}=\sum_{j=1}^{n}\left({CB}_{j}\;x\;{PPC}_{j}\right)/n \end{array}$$

where *i* = the TPB factor *i*; *j* = the product composite item *j*; *n* = the number of items for AT, SN, or PBC.

The data of all the intention variables and TPB factors turned out to be skewed, and thus, logistic regression models were applied to explain the association between the factors and intention. For the logistic regression analyses, intention toward each of the measures against HPAI was divided into two categories based on the Likert scores given for each intention to identify respondents with low or high-level intentions. The responses “strongly disagree,” “disagree,” and “neutral” were considered to indicate a low-level intention to implement the measure, and the responses “agree” and “strongly agree” were considered to indicate a high-level intention to implement the measure [(28), p. 4,633–4,634; (15), p. 141–142]. The TPB factors were categorized into three levels, based on the distribution of product composites score, in order to identify respondents who had weak, moderate, or strong AT, SN, and PBC. Theory of Planned Behavior factors (i.e., AT, SN, and PBC) that score <12 were considered weak; TPB factors that score ≥ 12 but <16 were considered moderate, and those scoring ≥16 were considered strong. If the number of observations in a category was smaller than 15, that category was merged with the nearest other category, resulting in two categories, that is, weak and moderate or moderate and strong. For all the logistic regression analyses using the TPB framework, the categorized intention variables were used as dependent variables, and the categorized AT, SN, and PBC factors were used as independent variables (i.e., one model for each intention). First, a univariable analysis was carried out to check the association between farming scheme and intentions. In this univariable analysis, farming scheme was used as fixed effect, and intention as a dependent variable; however, the analysis did not indicate any associations. Thus, farming scheme was excluded as independent variable in the logistic regression models.

For each of the four control measures, the following univariable and multivariable analyses were carried out. Before conducting multivariable logistic regression, univariable analysis was carried out to examine the association of each TPB factor with intention separately. Theory of Planned Behavior factors with a *p* < 0.25 were included in the multivariable logistic regression (29). Before conducting multivariable analyses, the presence of multicollinearity between TPB factors was checked using Spearman rank correlation coefficients (ρ). The multicollinearity check did not indicate high levels of correlation between TPB factors; all the correlation coefficients (ρ) were <0.8 [(30), p. 224]. Thus, all TPB factors were included in the multivariable analyses.

To evaluate the association of farmers' and farm characteristics with TPB factors, all the background information was included as binary variables in the multivariable logistic regression models. Farmers' and farm characteristics were used as independent variables, whereas TPB factors were used as dependent variables. Independent variables with a *p* < 0.15 by χ^2^ test, or by Fisher exact test when there were fewer than five observations in a cell, were included in the multivariable logistic regression analysis.

All multivariable logistic regression analyses were carried out using a backward stepwise procedure. Independent variables that were not significant (*p* > 0.05) were excluded from the models one-by-one at each step. All the statistical analyses were performed using SPSS version 25.0 (31).

## Results

### Descriptive Statistics

Table 2 provides the descriptive statistics of the sociodemographic and informational background of small-scale broiler farmers interviewed in this study. Of 203 small-scale broiler farmers interviewed, 141 (70%) were *makloon* farmers, 58 (28%) were contract farmers, and four (2%) were independent farmers. On average, participating small-scale broiler farmers were 45 years old, had 10 years of broiler farming experience, and had 3,000 birds on their farm. More than 75% depended on broiler farming activities as their main source of income.

**Table 2:** Descriptive statistics of sociodemographic characteristics and informational background of small-scale broiler farmers interviewed in this study (*n* = 203).

**Sociodemographic characteristics:**	**Freq. (*n*)**	**Percentage (%)**
**GENDER**		
Female	18	8.9
Male	185	91.1
**AGE**		
<45 years	100	49.3
≥45 years	103	50.7
**POSITION**		
Farm owner	180	88.7
Farm staff	23	11.3
**HIGHEST EDUCATION LEVEL**		
Elementary	70	34.5
Junior high school	66	32.5
Senior high school and higher education	67	33
**INCOME CONTRIBUTION FROM BROILER FARMING**		
25–50%	20	9.9
51–75%	67	33
>75%	105	51.7
N.A.	11	5.4
**INFORMATIONAL BACKGROUND OF FARMERS**		
Broiler farming experience		
≤10 years	128	63.1
>10 years	75	36.9
**CHICKEN POPULATION**		
≤3,000 birds	95	46.8
>3,000 birds	108	53.2
**KNOWLEDGE ABOUT THE EXISTENCE OF AVIAN INFLUENZA**		
Yes	155	76.4
No	48	23.6

Table 3 shows the descriptive statistics of intentions to implement HPAI measures. A large proportion of small-scale broiler farmers had a high level of intention to implement C&D (89%), vaccination (80%), and reporting (88%), while a smaller proportion of broiler farmers had a high level of intention for joining stamping-out with (67%) and without (53%) and compensation.

**Table 3:** Intentions of broiler farmers interviewed toward implementation of different measures to control HPAI.

**Measures**	**Statements** ***If within 1 year I know that HPAI exists within the subdistrict of my farm and risk my farm to get infected, therefore…***	***N***	**1** **SD**	**2** **D**	**3** **N**	**4** **A**	**5** **SA**	**High intenders (%)^a^**
Cleaning and disinfection (C&D)	I will clean and disinfect the barn every 2 days	203	0.5	3.4	7.4	57.6	31.0	89
AI vaccination	I will vaccinate my chickens on the seventh day in every cycle	180	0	6.1	13.9	55.0	25.0	80
Reporting	I will report to the technical support/vet as quick as possible if I observe one of my chickens showing the symptoms infected by AI	203	0	3.9	7.9	55.7	32.5	88
Stamping-out (no compensation)	I will join stamping-out if my farm were found to have an AI out even though I am not given any compensation	173	2.3	17.3	27.2	47.4	5.8	53
Stamping-out (50% compensation)	I will join stamping-out if my farm were found to have an AI out , and I am given 50% compensation for my healthy chickens that would be culled	173	0.6	6.9	26.0	53.8	12.7	67

a *Sum percentage of responses with scores 4 (agree) and 5 (strongly agree) for each intention variable*.

*SD, strongly disagree; D, disagree; N, neutral; A, agree; SA, strongly agree*.

Table 4 shows descriptive statistics and Cronbach α's of the categorized AT, SN, and PBC for each intention. Most broiler farmers (>80%) had a strong SN to the opinions of the referents to implement HPAI prevention and control on their farms. More than half of broiler farmers had a strong AT toward all intentions. In contrast, a relatively low proportion of broiler farmers had strong perceived control over their time and money investments toward C&D, vaccination, and stamping-out.

**Table 4:** Descriptive statistics and Cronbach α of categorized attitude (AT), subjective norm (SN), and perceived behavioral control (PBC) for all the intentions.

**Measures**	**Variables (AT, SN, PBC)^a^**	***n***	**Cronbach's** **α**	**Weak^b^** **(%)**	**Moderate^c^** **(%)**	**Strong^d^** **(%)**
Cleaning and disinfection/ sanitation	1. AT (sanitation)	203	0.792	9.4	30.5	60.1
	2. SN^e^					
	i. SN (technical support)	197		—	1.5	98.5
	ii. SN (vet nucleus)	180		—	2.3	97.8
	iii. SN (vet govt.)	170		—	4.7	95.3
	iv. SN (technical support medicine)	155		26.7	—	83.2
	v. SN (friends and role model farmers)	194	0.702	24.7	9.8	65.5
	3. PBC (money)	180		45.6	20.6	33.9
	4. PBC (time)	203		49.3	6.9	43.8
	5. PBC (skill)	203		91.6	8.4	—
Vaccination	1. Attitude (AT)	180	0.863	10.6	23.3	66.1
	2. PBC (money and time)	180	0.718	38.3	27.8	33.9
	3. PBC (skill)	195		63.6	36.4	—
Reporting	1. AT (morbidity and mortality)	203	0.803	—	40.4	59.6
	2. AT (stamping-out risk)	203		14.3	25.1	60.6
Stamping-out (no compensation)	1. AT stamping-out^f^	173	0.809	—	32.4	67.6
	2. PBC (money and time)	173	0.744	54.9	26	19.1
Stamping-out (50% compensation)	1. PBC (money and time)	173	0.709	56.6	25.4	17.9

a *AT, attitude; SN, subjective norm; PBC, perceived behavioral control*.

b *Weak category: AT, SN, or PBC scores <12*.

c *Moderate category: AT, SN, or PBC scores between 12 and <16*.

d *Strong category: AT, SN, or PBC scores ≥16*.

e *All subjective norms variables were included in univariable analyses for all the five intentions. The significant subjective norm variables were included in multivariable analyses for all the five intentions*.

f *Attitude stamping-out was used for both intentions to join stamping-out with no and 50% compensation*.

### TPB Factors in Relation to Intentions

Table 5 shows the statistically significant TPB factors for both the univariable and multivariable models for all intentions. Tables with detailed results for all the measures are provided in Appendices 1–5. The Nagelkerke *R*^2^ scores suggest that the intentions to implement those HPAI measures that are within the prime interest of broiler farmers (i.e., sanitation and vaccination) are explained better by the model, compared to the intentions for HPAI measures that are more important for public interest (i.e., reporting and stamping-out).

**Table 5:** Univariable and multivariable logistic regression model results showing the significant TPB factors for each of prevention, monitoring, and control measures against HPAI.

**Variables**	**Routine C&D**	**AI vaccination**	**Reporting**	**Stamping-out** **(no compensation)**	**Stamping-out** **(compensation)**
Attitude (AT)	<0.01^a^^,^^b^	<0.01^a^^,^^b^	n.s.	n.s.	n.s.
**Subjective Norms (SN)**					
Farmers	n.s.	n.s.	n.s.	n.s.	n.s.
TS	n.s.	n.s.	n.s.	n.s.	n.s.
Vet nucleus	n.s.	<0.05^a^; <0.01^b^	n.s.	n.s.	n.s.
Vet govt.	n.s.	n.s.	n.s.	n.s.	n.s.
TS medicine	n.s.	n.s.	n.s.	<0.05^a^	n.s.
**PERCEIVED BEHAVIORAL CONTROL (PBC)**					
Money	<0.01^a^	^−−^	n.a.	^−−^	^−−^
Time	<0.01^a^	^−−^	n.a.	^−−^	^−−^
Skill	n.s.	n.s.	n.a.	n.a.	n.a.
Money and time^c^	^−−^	<0.01^a^^,^^b^	n.a.	<0.01^a^^,^^b^	n.s.
***R***^**2**^	0.51	0.47	^−−^	0.17	^−−^

a *Univariable model*.

b *Multivariable model*.

c *Money and time factors were grouped together into a PBC factor (Cronbach α >0.7)*.

*n.s., not significant; n.a., not applicable*.

From our models, the AT factor was significantly (*P* < 0.05) and positively related to the intention of broiler farmers to implement routine C&D [odds ratio (OR), 211] and AI vaccination (OR, 20.4). The opinion of veterinarians of the integrated company (OR, 106.1) and the technical adviser from animal health companies (OR, 2.88) were significantly (*P* < 0.05) and positively associated to the intention of broiler farmers to implement AI vaccination and to join stamping-out without any compensation, respectively. The money and time factor were significantly (*P* < 0.05) and positively related to the intention of broiler farmers to implement routine C&D (money: OR, 14.5; time: OR, 9.8), AI vaccination (money and time: OR, 38.4), and to join stamping-out without any compensation (OR, 8.2). None of the TPB factors was significantly (*P* < 0.05) associated with the intention to join stamping-out with 50% compensation in either the univariable or multivariable model.

### Farmer and Farm Characteristics in Relation to Significant TPB Factors

Seven sociodemographic farmer characteristics, namely, age, gender, education, poultry farming experience, chicken population per cycle, awareness of HPAI and its signs, and dependency level on broiler farming, were regressed to TPB factors that were statistically significant in the models. Of these seven characteristics, only the contribution of broiler farming to farmers' income was found to be significantly associated with a strong AT toward the intentions to implement C&D and vaccination (Table 6). Farmers with a household income derived for 75% or more from broiler farming were more likely to have a strong AT toward C&D (OR, 7.3) and vaccination (OR, 20.2).

**Table 6:** Multivariate logistic regression model results describing the association of sociodemographic characteristics of broiler farmers with TPB factors that were significantly associated with the intentions.

**Measures**	**Background factors**	**TPB factors**	**Level**	**OR (95% CI)**
Cleaning and disinfection (C&D)	Income contribution	Attitude	≥75%	12.1 (2.11–69.39)^**^
			50–<75%	4.55 (0.78–26.61)
			25–<50%	ref.
Vaccination	Income contribution	Attitude	≥75%	20.24 (3.48–117.71)^**^
			50–<75%	2.36 (0.52–10.61)
			25–<50%	ref.

*^a^Odds ratio*.

*^b^Reference category*.

*^c^95% confidence interval*.

## Discussion

This study was carried out to gain insight into the psychological factors that determine intentions of small-scale broiler farmers to implement measures against HPAI and to identify sociodemographic characteristics associated with these psychological factors. We applied the TPB that states behavioral intention as a proxy measure of an actual implementation of behavior (14). Our study has several limitations. First, we had an inadequate number of respondents from the group of independent farmers. During the field work, we received information from government officials and other respondents that many independent farmers have closed down their farms or changed to rear male layer chickens. This is because small-scale independent broiler farmers have been experiencing financial losses due to the low market prices of live broiler chickens. In our survey, we tried to get as many independent farmers as possible given the time we had available. The number of independent farmers that we were able to interview was lower than planned. As a consequence, we had more respondents that were price-contract or makloon farmers. Second, in this study, we faced problems with retrieving data on disease or outbreak status of farms, similar to another study that was conducted in the same region (13). The unavailability of the data is because record-keeping is not usual on these farms, and farmers are not aware of the clinical signs of HPAI; hence, only severe HPAI outbreaks will be reported. As a result, information about HPAI is less obvious and trustful. To make sure that farms that had an outbreak in the past were included in our study, we took a pragmatic approach of sampling, taking into account the involvement of broiler farms that experienced a disease outbreak. Lastly, our study generated a more general understanding of the factors that determine the motivation of broiler farmers, compared to other studies that focus on a specific measure [e.g., (32)]. Since we looked at multiple measures, it was not feasible to probe in extensive detail for each of them in our survey, especially with regard to biosecurity measures that comprise various kinds of measures targeted for different HPAI introduction pathways. However, having four different measures in one study allowed us to identify similarities and differences in factors that influence the motivation of farmers for different measures.

More than 80% of the small-scale broiler farmers were motivated to take up preventive and monitoring measures, such as C&D, vaccination, and reporting. A lower number of small-scale broiler farmers had motivation to join stamping-out either with or without compensation. Our findings suggest that broiler farmers are more willing to take up measures that support their own interests compared to the public interest. The motivation of broiler farmers to implement preventive measures is in line with the preference of the Indonesian government for a vaccination-based HPAI mitigation strategy [(9), p. 7–8].

A large proportion of broiler farmers had a strong AT toward the intentions to implement regular C&D on the farm and vaccination. These findings suggest that broiler farmers had strong beliefs and placed high priorities on the benefits of improving the sanitation of their farm and of implementing AI vaccination for protection of both their poultry and humans from HPAI. The strong AT toward both measures was more likely for broiler farmers who have broiler farming as their main occupation.

For SN, our study showed that only the opinion of veterinarians of the integrated company positively influenced broiler farmers' intentions to vaccinate their chickens. This finding suggests that broiler farmers have a strong belief and MC with the opinion of the veterinarians regarding prevention and control. Clearly, the result came from respondents who were mostly either price-contract or makloon farmers. For independent farmers, TS or veterinarians from an animal health company may replace the role of veterinarians of the integrated company to influence farmers' decision to implement AI vaccination on their farm. However, our findings on SN were based only on a general level (e.g., prevention and control) rather than specifically directed to the measures (e.g., vaccination). Questions or statements that specifically evaluate the opinions of others about a specific measure against AI might better explain the effect of SN on the intention of farmers to implement specific measures.

For PBC, being in control of the consequences for time and income significantly determined the intentions of farmers to implement C&D measures and AI vaccination. Perceived behavioral control is more likely to determine the intentions of broiler farmers when they have less control over the consequences of implementation. For example, the OR of PBC for vaccination is higher than the PBC score for C&D. In this case, PBC might serve as a safety net for broiler farmers in case the consequences of the implementation of a measure are uncontrollable or beyond the expectation of the farmers. The significant associations between PBC and intentions also suggest that broiler farmers perceive those measures as costly and laborious. Despite this perception, they are still willing to spend their money and time on their implementation. Thus, broiler farmers who have more financial resources and spare time are more likely to clean and disinfect their farm more frequently and to vaccinate their chickens.

The significant association between AT and the intention to implement preventive measures suggests that there is room to develop relevant informational policy instruments. Informational instruments, such as providing practical information, could increase the internal motivation of farmers to perform specific behaviors [(32), p. 118]. To increase the adoption of HPAI control measures, practical information should be suited to the local context and promote financial benefits from implementation [(33), p. 530; (34), p. 11–12]. Training programs about prevention and control of HPAI, can be used by the government to increase the knowledge and awareness about HPAI and measures to control it. Training programs are already a priority supportive measure of the Indonesian government, but it is unclear how effective these programs are to actually increase knowledge and awareness about HPAI. The dissemination of the information in the training, in this case, should be done via credible communicators who are perceived by broiler farmers to be trustworthy and to have a high level of “similarity” [(32), p. 118], using education and communication materials developed by veterinarians, both public and private, and small-scale broiler farmers [(33), p. 530].

For HPAI mitigation in Indonesia, veterinarians of the integrated company would be better communicators than government-hired veterinarians. Because of high turnover, veterinarians hired by the government usually have less experience in working and communicating with broiler farmers compared to veterinarians from large integrators. Thus, the latter are more appropriate communicators when it comes to HPAI mitigation because they usually have more field experience and, more importantly, understand the local context better than government-hired veterinarians. Local governments could strengthen the communication with broiler farmers through farmer extension services, for instance, a periodic training and assistance program that are targeted to farmers and farm workers. This suggests that local governments should extend their current public–private partnership program by involving veterinarians from large integrators to raise the awareness of the importance of preventive measures among small-scale broiler farmers.

The finding that economic and time factors influence broiler farmers' intention to implement routine C&D and vaccination suggests that financial incentives and time-saving prevention and control scenarios will increase broiler farmers' motivation to implement those measures. These are basically economic elements. Furthermore, our study also found that the more broiler farmers are dependent on their broiler farm as their main source of income, the more likely they have positive AT toward routine C&D and vaccination measures to prevent HPAI. These findings suggest that programs that incentivize broiler farmers to increase their biosecurity and vaccinate their chickens may be helpful. Financial incentives in the form of a bonus on the market price and/or on performance could be applied as instruments to increase the uptake and the continuity of the implementation of biosecurity [(35), p. 599]. The same incentive could also be applied for vaccinated chickens. Moreover, vaccination may also be stimulated by reducing the costs of vaccination by a subsidized vaccine or assistance with vaccination, as is done for backyard poultry farms. However, because we do not know the economic impact of routine C&D and vaccination, we could not identify the exact incentives that would be suitable for broiler farmers in this case. Further research is needed to identify appropriate economic incentives for broiler farmers who implement vaccination and biosecurity measures on their farm.

## Conclusions

This study clarifies that small-scale broiler farmers are more in favor of preventive measures compared to monitoring and control measures directed against HPAI. Furthermore, our findings suggest that factors, such as broiler farmers' AT, opinions of veterinarians of nucleus company, and broiler farmers' financial and time resources, were positively associated with one or more of broiler farmers' intentions to implement preventive and control measures against HPAI. Our results also suggest that informational and financial instruments are appropriate instruments to increase the uptake of prevention and control measures by small-scale broiler farmers and could help mitigate HPAI spread in Western Java.

## Data Availability Statement

The datasets generated for this study are available on request to the corresponding author.

## Ethics Statement

Ethical review and approval was not required for the study on human participants in accordance with the local legislation and institutional requirements. The patients/participants provided their written informed consent to participate in this study.

## Author Contributions

MP designed the study, collected and analyzed the data, and drafted the manuscript. The remaining authors provided input on the design of the study, helped in interpreting study results, and critically revised the manuscript. All authors read and approved the final manuscript. All authors contributed to the article and approved the submitted version.

## Conflict of Interest

The authors declare that the research was conducted in the absence of any commercial or financial relationships that could be construed as a potential conflict of interest.
