# Supplementary material for: Understanding the Motivation of Western Java Smallholder Broiler Farmers to Uptake Measures Against Highly Pathogenic Avian Influenza (HPAI)
Source: Front Vet Sci. 2020 Jul 21;7:362. doi: 10.3389/fvets.2020.00362 (PMC7394697; doi:10.3389/fvets.2020.00362)
Supplement: Supplementary file 1 [file Data_Sheet_1.pdf]

Supplementary Material

Understanding the Motivation of Western Java Smallholder Broiler Farmers to Uptake Measures Against Highly Pathogenic Avian Influenza (HPAI)

Muchammad Gumilang Pramuwidyatama*, Henk Hogeveen, Helmut Willem Saatkamp

*** Correspondence:** Muchammad Gumilang Pramuwidyatama: muchammad.gumilang@wur.nl

1 Appendices

Appendix 1. Univariable and multivariable logistic regression model results showing the association of broiler farmers' attitude, subjective norm, and perceived behavioral control with intention to implement C&D of farm area and barn once in every two days.

Variables	Univariable model				Multivariable model (R^{2b}= 0.51)			
	Weak	Moderate	Strong	p^a	Weak	Moderate	Strong	p
	Odds ratio (95% CI)				Odds ratio (95% CI)			
**1. AT**	ref.	1.92 (0.61-6.1)	10.8 (2.9-40.34)**	**0.001**	ref.	21.94 (1.44-333.95)*	211.28 (11-4055.68)**	0.001
**2. SN**								
Farmers	ref.	1.4 (0.34-5.8)	3.08 (1.2-7.96)*	**0.063**				
TS		ref.	3.9 (0.34-44.9)	0.274				
Vet nucleus		ref.	<0.0001	0.999				
Vet govt		ref.	<0.0001	0.999				
TS medicine		ref.	0.63 (0.14-2.95)	0.561				
**3. PBC**								
Money	ref.	8.73 (1.11-68.52)*	14.55 (1.87-113.02)*	**0.006**	ref.	15.18 (1.16-198.66)*	4.49 (0.166-121.76)	0.108
Time	ref.		9.82 (2.24-43.13)**	**0.002**	ref.		27.36 (0.86-869.37)	0.061
Skill	ref.	2.15 (0.27-16.98)		0.46				

AT= Attitude, SN= Subjective norm, PBC= Perceived behavioral control; OR= odds ratio; CI= confidence interval
* significant at $p<0.05$; ** significant at $p<0.01$.
[a]Variables with p value lower than 0.25 in the univariable model were given in bold and included in the multivariable model.
[b]Nagelkerke R^2.

Appendix 2. Univariable and multivariable logistic regression model results showing the association of broiler farmers' attitude, subjective norm, and perceived behavioral control with intention to implement AI vaccination to their chickens on the seventh day in every production cycle.

**Variables**	**Univariable model**				**Multivariable model (R^{2b}= 0.47)**			
	**Weak**	**Moderate**	**Strong**	***p*[a]**	**Weak**	**Moderate**	**Strong**	***p***
	**Odds ratio (95% CI)**				**Odds ratio (95% CI)**			
**1. AT**	ref.	1.17 (0.37-3.62)	4.04 (1.38-11.89)*	**0.004**	ref.	4.76 (0.78-29.15)	20.43 (3.44-121.43)**	0.001
**2. SN**								
Farmers	ref.	1.3 (0.25-7.25)	0.97 (0.39-2.37)	0.924				
TS	ref.		412712551.1	0.999				
Vet nucleus	ref.		12 (1.21-119.22)*	**0.034**	ref.		106.11 (5.47-2059.99)**	0.002
Vet govt	ref.		<0.0001	0.999				
TS medicine	ref.		<0.0002	0.998				
**3. PBC**								
Money & time	ref.	10.7 (3.03-37.8)**	7.65 (2.72-21.5)**	**<0.001**	ref.	34.94 (5.32-229.62)**	38.39 (6.16-239.14)**	<0.001
Skill	ref.	2.02 (0.89-4.62)		**0.094**				

AT= Attitude, SN= Subjective norm, PBC= Perceived behavioral control; OR= odds ratio; CI= confidence interval

* significant at $p<0.05$; ** significant at $p<0.01$.

[a]Variables with *p* value lower than 0.25 in the univariable model were given in bold and included in the multivariable model.

[b]Nagelkerke R^2.

Appendix 3. Univariable and multivariable logistic regression model results showing the association of broiler farmers' attitude and subjective norm with intention to report an incidence/outbreak of AI on the farm to the authorities.

**Variables**	**Univariable model**				**Multivariable model**			
	**Weak**	**Moderate**	**Strong**	p^a	**Weak**	**Moderate**	**Strong**	p
	**Odds ratio (95% CI)**				**Odds ratio (95% CI)**			
**1. AT**								
Morbidity & mortality		ref.	0.57 (0.23-1.45)	**0.237**				
Selective depopulation risk	ref.	<0.0001	<0.0001	0.622				
**2. SN**								
Farmers	ref.	4.74 (0.56-38.89)	2.52 (1.01-6.3)*	**0.088**				
TS		ref.	<0.0001	0.999				
Vet nucleus		ref.	<0.0001	0.999				
Vet govt		ref.	<0.001	0.999				
TS medicine		ref.	0.36 (0.05-2.86)	0.332				

AT= Attitude, SN= Subjective norm, PBC= Perceived behavioral control; OR= odds ratio; CI= confidence interval
* significant at $p<0.05$; ** significant at $p<0.01$.
[a]Variables with p value lower than 0.25 in the univariable model were given in bold and included in the multivariable model.

Appendix 4. Univariable and multivariable logistic regression model results showing the association of broiler farmers' attitude, subjective norm, and perceived behavioral control with intention to join stamping-out without any compensation in case of an HPAI outbreak on the farm.

**Variables**	**Univariable model**				**Multivariable model (R^{2b}= 0.17)**			
	**Weak**	**Moderate**	**Strong**	***p*[a]**	**Weak**	**Moderate**	**Strong**	***p***
	**Odds ratio (95% CI)**				**Odds ratio (95% CI)**			
**1. AT**		ref.	1.49 (0.79-2.83)	**0.219**				
**2. SN**								
Farmers	ref.	0.27 (0.06-1.2)	0.69 (0.33-1.45)	**0.212**				
TS		ref.	1.12 (0.07-18.13)	0.94				
Vet nucleus		ref.	1974468944	0.999				
Vet govt		ref.	1.09 (0.26-4.5)	0.911				
TS medicine		ref.	2.88 (1.14-7.25)*	**0.025**				
**3. PBC**	ref.	2.7 (1.3-5.6)**	9.18 (3.25-25.9)**	**<0.001**	ref.	2.61 (1.25-5.43)*	8.23 (2.89-23.4)**	<0.001

AT= Attitude, SN= Subjective norm, PBC= Perceived behavioral control; OR= odds ratio; CI= confidence interval
* significant at p<0.05; ** significant at p<0.01.
[a]Variables with *p* value lower than 0.25 in the univariable model were given in bold and included in the multivariable model.
[b]Nagelkerke R^2.

Appendix 5. Univariable and multivariable logistic regression model results showing the association of broiler farmers' attitude, subjective norm, and perceived behavioral control with intention to join stamping-out with 50% compensation in case of an HPAI outbreak on the farm.

**Variables**	**Univariable model**				**Multivariable model**			
	**Weak**	**Moderate**	**Strong**	***p***[a]	**Weak**	**Moderate**	**Strong**	***p***
	**Odds ratio (95% CI)**				**Odds ratio (95% CI)**			
**1. AT**		ref.	1.46 (0.75-2.83)	0.268				
**2. SN**								
Farmers	ref.	1.04 (0.23-4.71)	0.86 (0.39-1.86)	0.905				
TS		ref.	2.1 (0.13-34.4)	0.599				
Vet nucleus		ref.	6.8 (0.69-66.98)	**0.101**				
Vet govt		ref.	0.29 (0.036-2.49)	0.263				
TS medicine	ref.		1.25 (0.5-3.11)	0.635				
**3. PBC**	ref.	1.75 (0.83-3.7)	1316312835	0.345				

AT= Attitude, SN= Subjective norm, PBC= Perceived behavioral control; OR= odds ratio; CI= confidence interval
* significant at $p<0.05$; ** significant at $p<0.01$.
[a]Variables with *p* value lower than 0.25 in the univariable model were given in bold and included in the multivariable model.
